# Clinical relevance of bacterial and/or viral coinfection in acute bronchiolitis in an Italian neonatal unit during the 2021–2023 seasons

**DOI:** 10.3389/fped.2025.1577913

**Published:** 2025-05-30

**Authors:** Venere Cortazzo, Marilena Agosta, Domenico Umberto De Rose, Valeria Fox, Velia Chiara Di Maio, Gianluca Vrenna, Martina Rossitto, Barbara Lucignano, Stefania Ranno, Annamaria Sisto, Cristina Russo, Annabella Braguglia, Maria Paola Ronchetti, Andrea Dotta, Carlo Federico Perno, Paola Bernaschi

**Affiliations:** ^1^ Microbiology and Diagnostic Immunology Unit, Bambino Gesù Children’s Hospital, IRCCS, Rome, Italy; ^2^ Neonatal Intensive Care Unit, Bambino Gesù Children’s Hospital IRCCS, Rome, Italy; ^3^ PhD Course in Microbiology, Immunology, Infectious Diseases, and Transplants (MIMIT), Faculty of Medicine and Surgery, “Tor Vergata” University of Rome, Rome, Italy; ^4^ Multimodal Laboratory Medicine, Bambino Gesù Children’s Hospital, IRCCS, Rome, Italy; ^5^ Neonatal Sub-Intensive Care Unit and Follow-up, Bambino Gesù Children’s Hospital IRCCS, Rome, Italy

**Keywords:** newborns, bronchiolitis, respiratory infections, coinfections, infants, viral monoinfection, RSV, rhinovirus

## Abstract

**Background:**

Acute bronchiolitis is a leading cause of hospitalization in young children worldwide, and literature reports conflicting data regarding the role of coinfections.

**Objective:**

To evaluate the possible clinical relevance of bacterial and/or viral respiratory coinfection in a cohort of newborns/infants hospitalized for bronchiolitis.

**Methods:**

Neonates and infants younger than three months admitted to neonatal units from October 2021 to March 2023 because of acute bronchiolitis were included in this retrospective study. Analyses were performed with Stata 11.1 (*p* < 0.05). Data were summarized as medians (IQR) or counts (%). Appropriate tests were used based on data type and distribution, with Benjamini–Hochberg correction for multiple comparisons. Odd Ratios (ORs) were unadjusted.

**Results:**

In a cohort of 240 patients, respiratory coinfection was associated with a longer hospital stay (*p* < 0.001) and the need for invasive mechanical ventilation (*p* < 0.001) compared to viral mono-infection, highlighting a potential role in patient outcome. Moreover, we observed that premature patients are more likely to contract a respiratory coinfection than a viral mono-infection (*p* = 0.011).

**Conclusion:**

Coinfections increased the clinical severity of bronchiolitis more than simple viral mono-infection in our cohort, contributing to a longer hospital stay and the need for invasive mechanical ventilation.

## 1 Introduction

Respiratory tract infections represent a significant public health problem due to limited therapeutic arsenal and emerging therapeutic resistance. Among respiratory infections, acute bronchiolitis is a common lung infection and a major cause of hospitalization in young children (1). In high-income countries, bronchiolitis accounts for up to 15%–17% of all hospitalizations in children younger than 2 years and 15% of emergency department presentations in infants (2).

Typically, signs of upper respiratory tract infection occur after an incubation period of 4–6 days. Subsequently, lower respiratory tract involvement becomes evident with variable degrees of breathing difficulty, crackles, and bilateral wheezing upon chest examination (2–4). The variable clinical presentation and the potential for sudden deterioration of the clinical conditions require close monitoring by healthcare professionals (5).

Respiratory syncytial virus (RSV) is the infective organism reported to be the most common cause of bronchiolitis (6). As viral detection methods are improving and their use is expanding, multiple viruses are increasingly found in infants with bronchiolitis. Indeed, other viruses associated with bronchiolitis, which often occur as coinfections, include rhinovirus, human metapneumovirus, adenovirus, parainfluenza viruses (PIVs 1–4), influenza viruses (Flu A/B), human bocavirus, enterovirus and human coronaviruses (7).

Non-viral coinfections with *Bordetella pertussis* and other atypical bacteria (i.e., *Mycoplasma pneumoniae*, as well as *Chlamydia pneumoniae* and *Chlamydia trachomatis*) or other bacteria are occasionally reported (2).

Despite growing interest, the clinical significance of viral and/or bacterial coinfections in bronchiolitis remains unclear, with conflicting evidence on their impact on disease severity, clinical course, and healthcare resource utilization (8–12). Therefore, this study aims to evaluate the clinical relevance of viral and bacterial coinfections in a cohort of newborns and infants hospitalized for bronchiolitis, to better understand their potential influence on patient management and outcomes.

## 2 Methods

### 2.1 Study design

We carried out a retrospective, observational study on newborns/infants under three months admitted to the Neonatal Intensive and Sub-Intensive Care Unit of Bambino Gesù Children's Hospital in Rome (Italy) for bronchiolitis from October 2021 to March 2023. Data were collected from electronic medical records, including demographic characteristics, laboratory findings, clinical outcomes, and treatments. Disease severity was classified based on respiratory support requirements. The diagnosis of bronchiolitis was based on clinical evaluation, according to national and international guidelines (1, 2). Typical criteria included signs of viral upper respiratory infection with increased respiratory effort (tachypnea, nasal flaring, chest retractions), wheezing, and/or crackles on auscultation in infants under 12 months. Disease severity was classified based on the level of respiratory support required, categorized as mild (no oxygen requirement), moderate (non-invasive oxygen support), or severe (invasive mechanical ventilation), consistent with current literature and clinical practice (1, 2).

### 2.2 Study population

This study included 240 neonates and infants (<3 months old) hospitalized with a clinical diagnosis of bronchiolitis. Mild cases not requiring hospitalization were excluded. Patients with congenital anomalies, primary immunodeficiencies, or other severe comorbidities were also excluded to ensure a more homogeneous study population.

### 2.3 Microbiology testing

All newborns/infants underwent standardized sample collection upon admission to the Emergency Department, and a microbiological investigation was carried out on samples from the upper respiratory tract for etiological diagnosis. The samples were processed and managed according to the standardized protocols of the Microbiology Laboratory.

Viral respiratory infection was defined by the detection of one or more viral etiological agents on any of the respiratory specimens, using molecular Polymerase Chain Reaction (PCR) methods (i.e., Allplex Respiratory Panel Assays [Seegene, Seoul, South Korea] or BioFire FilmArray Respiratory 2.1 Panel [BioMérieux Clinical Diagnostics, Salt Lake City, Utah, United States]). Bacterial infection was defined by a positive PCR result or a positive microbiological culture of the respiratory samples.

### 2.4 Ethics statements

The authors assert that all procedures of the study comply with the ethical standards of the institutional and national research committee and with the 1964 Helsinki Declaration and its later amendments or comparable ethical standards (13). Personal data were restricted to essential information and were treated in order to guarantee the respect of the privacy of the involved patients, as specifically stated by Italian Law D. Lgs. n.196 of 2003 about personal data protection. Written informed consent was not required, as the study is retrospective and has no patient-identifiable information. Despite this, our Scientific Directorate validated the study before submission to the journal, as in our hospital, all studies performed have to be approved by this office. In addition, parents or legal guardians of patients provided consent to use personal data for diagnosis, treatment, and related research purposes at the time of hospitalization.

### 2.5 Statistical analysis

Stata software version 11.1 (StataCorp, College Station, TX, USA) was used for statistical analysis, with significance set at *p* < 0.05. Descriptive statistics are expressed as median values and interquartile range (IQR) for continuous data and number (percentage) for categorical data. For categorical variables, significant differences were assessed by the Chi-square test (when all expected cell counts were ≥25) or Fisher Exact Test (if at least one cell had an expected count <25). For continuous variables, normality was assessed: if the data followed a normal distribution, comparisons between groups were conducted using the Student's *t*-test, otherwise the Mann–Whitney *U* test was applied. To adjust the significance for multiple comparisons and confirm potential associations, a Benjamini–Hochberg correction was applied. For odds ratio (OR) calculations, no logistic regression was used to adjust for potential confounders.

## 3 Results

### 3.1 Patients

**Figure 1:**
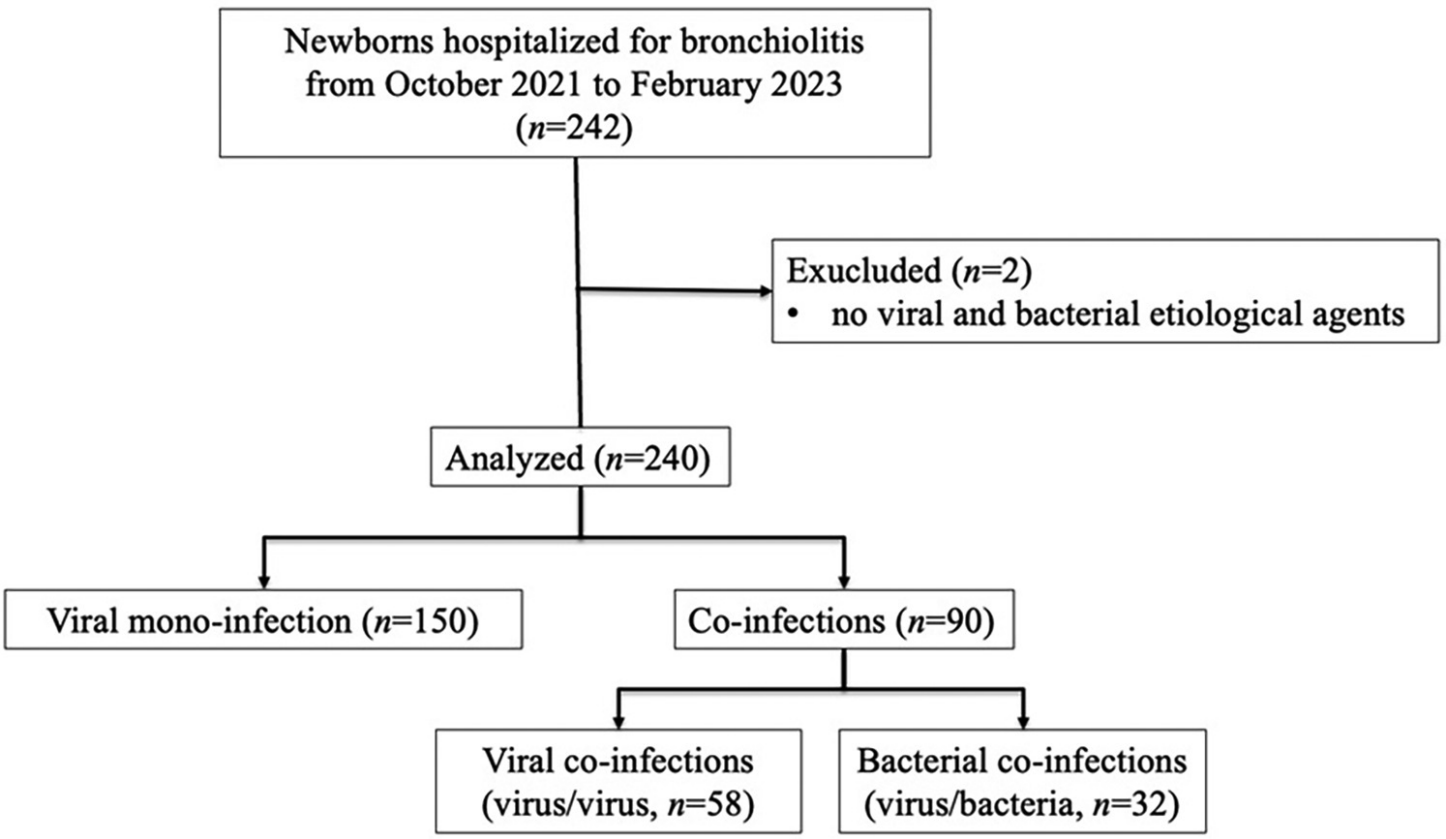
Flow-chart of patient inclusion.

From October 2021 to March 2023, 242 neonates and infants were admitted for bronchiolitis. The final study population consisted of 240 newborns/infants; two patients with no detected etiological agents were excluded, as shown in Figure 1.

Table 1 compares the demographic and clinical characteristics and diagnosis of the study population's monomicrobial or polymicrobial respiratory infections. In addition, the values representing the strength of the association between some independent variables and the presence of viral and/or bacterial coinfection (odds ratio) are shown in Table 2. All considerations regarding coinfection were independent of the etiology of the coinfection itself, whether with virus/virus or virus/bacteria detected, probably attributable to the cohort of patients analyzed in this study.

**Table 1:** Comparison of demographic and clinical characteristics of newborns/infants hospitalized with monomicrobial or polymicrobial respiratory infections.

	Mono-infections	Coinfections	*p* value
240 newborns/infants [no. (%)]	150 (62.5)	90 (37.5)	
Age (days) [median (IQR)]	22.5 (15–30.8)	29 (21–41)	**<0** **.** **001** *
Male [no. (%)]	74 (49.3)	36 (40)	0.182
Breastfeeding [no. (%)]	128 (85.3)	74 (82.2)	0.585
Sick relatives at home [no. (%)]	102 (68.0)	66 (73.3)	0.467
Length of hospital stay (days) [median (IQR)]	5 (3–8)	7 (5–12)	**<0** **.** **001** #
Prematurity/late preterm [no. (%)]	6 (4.0)	12 (13.3)	**0** **.** **011** *
Previous Palivizumab prophylaxis [no. (%)]	1 (0.7)	3 (3.3)	0.150
Other pre-existing co-morbidity [no. (%)]	5 (3.3)	3 (3.3)	1.000
Fever [no. (%)]	26 (17.3)	18 (20.0)	0.609
Invasive mechanical ventilation [no. (%)]	1 (0.7)	14 (15.6)	**<0** **.** **001** #
No-invasive ventilation [no. (%)]	124 (82.7)	75 (83.3)	1.000
Duration of respiratory assistance (days) [median (IQR)]	5 (3–7)	5 (3–9)	0.072
Laboratory findings at time of diagnosis [median (IQR)]			
Procalcitonin level (ng/ml) [median (IQR)]	0.16 (0.12–0.34)	0.22 (0.14–0.22)	0.486
C-reactive protein level (mg/dl) [median (IQR)]	0.46 (0.1–1.43)	0.56 (0.20–1.70)	0.375
White blood cell count/mm3 [median (IQR)]	10,010 (8,060–12,410)	10,260 (7,925–12,383)	0.422
% Neutrophil [median (IQR)]	34.7 (23.9–46.2)	37.3 (26.8–47.08)	0.149
% Lymphocyte [median (IQR)]	46 (36.1–55)	43.5 (35.1–53.2)	0.135
Episodes with concurrent bacteremia [no. (%)]	3 (2.0)	3 (3.3)	0.674
Episodes with concurrent non-invasive bacterial infections [no. (%)]	17 (11.3)	15 (16.7)	0.246

Data are expressed as *n* (%) or median (IQR).

* Two-sided *p*-values were calculated by Chi-square test, or Mann–Whitney test, as appropriate.

# *p*-values that remain significant even after the Benjamini-Hochberg correction. Bold values are statistically significant differences (*p* < 0.05).

**Table 2:** Values representing the strength of the association between independent variables and the presence of viral and/or bacterial coinfection*.*

	Monoinfections	Coinfections	*p* value	OR (95% CI)
Male [no. (%)]	74 (49.3)	36 (40)	0.182	1.461 (0.860–2.480)
Breastfeeding	128 (85.3)	74 (82.2)	0.585	1.258 (0.622–2.545)
Sick relatives at home	102 (68.0)	66 (73.3)	0.467	0.773 (0.433–1.380)
Prematurity/late preterm [no. (%)]	6 (4.0)	12 (13.3)	0.011*	0.271 (0.098–0.750)
Other pre-existing co-morbidity [no. (%)]	5 (3.3)	3 (3.3)	1.000	1.000 (0.233–4.288)
Fever [no. (%)]	26 (17.3)	18 (20.0)	0.609	0.839 (0.430–1.635)
Invasive mechanical ventilation [no. (%)]	1 (0.7)	14 (15.6)	**<0.001**#	0.036 (0.005–0.282)
No-invasive ventilation [no. (%)]	124 (82.7)	75 (83.3)	1.000	0.954 (0.475–1.915)
Episodes with concurrent bacteremia [no. (%)]	3 (2.0)	3 (3.3)	0.674	0.592 (0.117–2.997)
Episodes with concurrent non-invasive bacterial infections [no. (%)]	17 (11.3)	15 (16.7)	0.246	0.639 (0.302–1.353)

Data are expressed as *n* (%).

* Two-sided *p*-values were calculated by Chi-square test.

#*p*-values that remain significant even after the Benjamini-Hochberg correction.

Bold values are statistically significant differences (*p* < 0.05).

### 3.2 Microbiology, laboratory and clinical results

One hundred and fifty (62.5%) patients had viral mono-infection, and 90 had coinfections (37.5%), as detailed: 58 (64.4%) had only viral coinfections (virus/virus), and 32 (35.6%) had both bacterial and viral coinfections (virus/bacteria). Bacterial coinfection in hospitalized patients was reported in 13.3% (32/240); in particular, the presence of a single bacterium was detected in 19 coinfections, and the presence of ≥2 bacteria in 13 coinfections.

Among 150 patients with viral respiratory mono-infection, the median age was 22.5 days (IQR, 15–30.8 days), and 74 were (49.3%) males. Among 90 patients with respiratory coinfection (virus/virus or virus/bacteria), the median age was 29 days (IQR, 21–41 days), and 36 were (40%) males. Patients with coinfections were significantly older than those with viral mono-infections (median age 29 vs. 22 days, *p* < 0.001), suggesting increased susceptibility with age.

**Figure 2:**
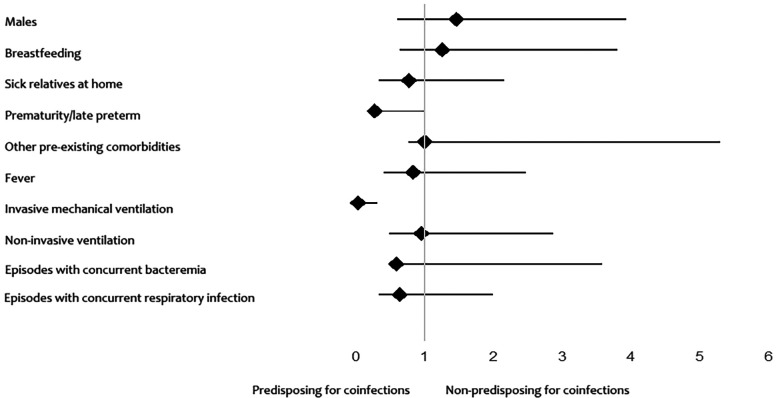
Associations with the risk of having a mono-infection and coinfection.

Premature patients had a significantly lower probability (73% reduction) of mono-infection than term patients, suggesting a higher susceptibility to respiratory coinfections. Patients who received invasive mechanical ventilation were significantly less likely (96.4% reduction) to have had a respiratory mono-infection. The two confidence intervals do not include 1, so these associations are statistically significant (Figure 2).

Infectious agents identified in all newborns/infants with bronchiolitis are reported in Table 3. As expected, RSV was the most common viral etiological agent identified in our study (82.5%), while rhinovirus was the second most common virus detected (23.8%). In all 240 samples analyzed, a total of 48 bacteria were detected (20%). *Haemophilus influenzae* (7.9%), *Moraxella catarrhalis* (3.8%), and *Streptococcus pneumoniae* (3.3%) were the three most commonly identified bacteria.

**Table 3:** Infectious agents identified in all patients (*n* = 240) with bronchiolitis during 2021–2023*.*

Infectious agent	Respiratory monoinfection	Respiratory coinfections (viral and/or bacterial)	Overall (%)	*p* value
Viruses				
RSV (no.)^a^	119	79	198 (82.5%)	**<0.001** *
Rhinovirus (no.)	11	46	57 (23.8%)	**<0.001** *
Human Metapneumovirus (no.)	8	7	15 (6.3%)	0.794
Parainfluenza (no.)	5	7	12 (5%)	0.771
Coronavirus OC43/229° (no.)	5	6	11 (4.6%)	1.000
Influenza A (no.)	2	6	8 (3.3%)	0.285
Adenovirus (no.)	0	3	3 (1.3%)	0.248
Enterovirus (no.)	0	3	3 (1.3%)	0.248
SARS-CoV-2 (no.)	0	2	2 (0.8%)	0.499
Bacteria				
*Haemophilus influenzae* (no.)	0	19	19 (7.9%)	/
*Moraxella catarrhalis* (no.)	0	9	9 (3.8%)	/
*Streptococcus pneumoniae* (no.)	0	8	8 (3.3%)	/
*Staphylococcus aureus* (no.)	0	7	7 (2.9%)	/
*Bordetella parapertussis* (no.)	0	2	2 (0.8%)	/
*Escherichia coli* (no.)	0	1	1 (0.4%)	/
*Pseudomonas aeruginosa* (no.)	0	1	1 (0.4%)	/
*Klebsiella pneumoniae* (no.)	0	1	1 (0.4%)	/

^a^ Among 198 RSV detected, 14 were typed as RSV–A, 41 as RSV–B, 64 were not typed.

Data are expressed as *n* (%).

* Two-sided *p*-values were calculated by Chi-square test.

# *p*-values that remain significant even after the Benjamini–Hochberg correction.

Bold values are statistically significant differences (*p* < 0.05).

Analyzing demographic and clinical characteristics of newborns/infants in case of viral or bacterial coinfections, we found a significantly lower neutrophil percentage and higher lymphocyte count in patients with viral coinfections (*p* = 0.038 and *p* = 0.029, respectively) (Table 4).

**Table 4:** Demographic and clinical characteristics of newborns/infants in case of viral or bacterial coinfections*.*

	Viral coinfections	Bacterial coinfections	*p* value
240 newborns/infants [no. (%)]	58 (64.4)	32 (35.6)	
Age (days) [median (IQR)]	28 (20.3–45.5)	31.5 (21–37.5)	0.243
Male [no. (%)]	26 (44.8)	10 (31.3)	0.263
Breastfeeding [no. (%)]	48 (82.8)	26 (81.3)	1.000
Sick relatives at home [no. (%)]	41 (70.7)	25 (78.1)	0.619
Length of hospital stay (days) [median (IQR)]	6 (4–8.8)	9.5 (6.8–12.2)	0.086
Prematurity/late preterm [no. (%)]	6 (10.3)	6 (18.8)	0.334
Previous Palivizumab prophylaxis [no. (%)]	2 (3.4)	1 (3.1)	1.000
Other pre-existing co-morbidity [no. (%)]	3 (5.2)	0 (0)	0.550
Fever [no. (%)]	11 (19.0)	7 (21.9)	0.787
Invasive mechanical ventilation [no. (%)]	6 (10.3)	8 (25.0)	0.077
No-invasive ventilation [no. (%)]	47 (81.0)	28 (87.5)	0.560
Duration of respiratory assistance (days) [median (IQR)]	5 (3–9)	6 (4–8)	0.164
Laboratory findings at time of diagnosis [median (IQR)]			
Procalcitonin level (ng/ml) [median (IQR)]	0.24 (0.16–0.32)	0.17 (0.13–0.2)	0.189
C-reactive protein level (mg/dl) [median (IQR)]	0.44 (0.19–1.19)	0.9 (0.31–2.28)	0.080
White blood cell count/mm3 [median (IQR)]	10,190 (7,843–12,358)	10,425 (8,020–12,748)	0.133
% Neutrophil [median (IQR)]	35.4 (24.05–46.65)	41.2 (32.9–48.7)	**0** **.** **038** *
% Lymphocyte [median (IQR)]	44.6 (36.05–54.88)	38.15 (33.55–46.18)	**0** **.** **029** *
Episodes with concurrent bacteremia [no. (%)]	1 (1.7)	2 (6.3)	0.287
Episodes with concurrent non-invasive bacterial infections [no. (%)]	9 (15.5)	6 (18.8)	0.771

Data are expressed as *n* (%) or median (IQR).

* Two-sided *p*-values were calculated by Chi-square test, or Mann–Whitney test, as appropriate.

None of the *p*-values remained significant even after the Benjamini–Hochberg correction. Bold values are statistically significant differences (*p* < 0.05).

## 4 Discussion

Bronchiolitis can be a severe cause of respiratory failure in newborns (14). Compared to viral mono-infection, patients with coinfection were older (*p* < 0.001), highlighting a greater risk of contracting polymicrobial respiratory infections than in those younger. The explanation could be attributable to the persistence of maternal antibodies that protect newborns from infections in the first weeks of life (15). Indeed, it is conceivable that passive immunity to (at least some) respiratory pathogens can be transferred to neonates by transplacental maternal antigen-specific IgG antibodies induced by maternal colonization or vaccination (16, 17).

While the typical length of pregnancy is between 39 and 41 completed weeks of gestation, annually, 15 million children are stillborn preterm (a condition defined as delivery before 37 weeks) (18). The risks of adverse outcomes for preterm newborns increase significantly with a decrease in gestational age (19). In our cohort, those newborns/infants requiring hospitalization with coinfection were mostly preterm born (*p* = 0.011). Indeed, the most consistently identified risk factor associated with progression to severe bronchiolitis includes a gestational age of less than 37 weeks (2). This suggests that prematurity may also be a risk factor for contracting a polymicrobial respiratory infection.

In all our patients, respiratory coinfection was associated with a longer hospital stay (*p* < 0.001) and invasive mechanical ventilation (*p* < 0.001) compared to viral mono-infection, highlighting a potential role in patient outcome. As previously described, coinfection is associated with more severe disease (9), and, in addition, in our study, the severity of coinfections was independent of the etiology of the coinfection itself.

No significant differences were found considering other demographic and clinical characteristics, as shown in Table 1.

Furthermore, our findings highlighted, as demonstrated by other studies, that serious bacterial infections (i.e., bacteremia and meningitis) or other non-invasive concomitant bacterial infections are extremely rare in children with bronchiolitis, thus confirming the current guidelines that do not support the routine use of antibiotics in these patients (20).

We observed that RSV was mostly found in mono-infections (*p* < 0.001), while Rhinovirus was more frequently detected in coinfections (*p* < 0.001). The reasons why some viruses cause mono-infection or coinfection remain still unclear; one hypothesis is that the mono- vs. coinfection is associated with competitive interactions at the host-pathogen level (21). All viral associations found in our study were non-significant, without recurring associations. This result is different from what Mandelia and colleagues reported, namely that direct or indirect interactions occur between specific viral pathogens (21). This difference could be associated with the selected cohort of patients analyzed and does not exclude that specific associations can be found in other clinical conditions. Indeed, in another recent publication of our group, some specific viral associations have been found in patients with other clinical characteristics (22).

All infants diagnosed with bacterial coinfection received antibiotic therapy. However, the administration of antibiotics did not significantly correlate with a reduction in the duration of hospitalization or the need for mechanical ventilation. These findings suggest that while antibiotics were given for treating bacterial infections, their impact on overall disease severity in bronchiolitis cases remained limited.

Finally, the retrospective nature of the study design and the potential for selection or information bias may have influenced the results and their interpretation, representing a limitation of the study. As this was an observational study, we could only establish associations, not causal relationships, between coinfections and disease severity. Unmeasured confounders, such as viral load or specific host immune responses, may have influenced our findings.

Given the inherent limitations of standard microbiological and clinical assessments in rapidly distinguishing viral from bacterial involvement and in quantifying disease burden, there is a clear need for more precise, non-invasive diagnostic tools. In this context, emerging molecular imaging approaches hold considerable promise. Innovative agents—such as enzyme-activatable near-infrared fluorescent probes and pathogen-targeted radiotracers—have demonstrated the ability to non-invasively visualize and differentiate between viral and bacterial lung infections, as well as to monitor therapeutic response in real time (23–25). While these technologies remain largely in preclinical or early clinical stages, their future integration into routine practice could significantly enhance diagnostic accuracy and support personalized management strategies for vulnerable pediatric populations.

## 5 Conclusion

In our cohort, approximately one-third of newborns/infants hospitalized with bronchiolitis (37.5%) had a coinfection.

While viruses have been identified as the predominant infectious agents, it is important to recognize bacteria's role in respiratory coinfections. RSV (82.5%) and *Haemophilus influenzae* (7.9%) were the most common viral and bacterial etiological agents identified, respectively. Rhinovirus was the second most common viral agent in 23.8% of the cases. We observed that the increased detection of RSV and Rhinovirus in mono-infections and coinfections, respectively, was significant.

Coinfection increased the clinical severity of bronchiolitis more than simple viral mono-infection, contributing to a longer hospital stay and the need for invasive mechanical ventilation, especially in these young patients.

Finally, a significant association has been identified between prematurity and the presence of respiratory coinfections, considering prematurity as a risk factor.

Our results may be useful in understanding the prevalence and distribution of infectious agents in neonatal and pediatric respiratory infections to guide diagnostic and therapeutic strategies, including appropriate antibiotic management. Prospective studies would be needed to confirm our findings and deepen the understanding of factors influencing susceptibility to respiratory infections in newborns and infants.

## Data Availability

The raw data supporting the conclusions of this article will be made available by the authors, without undue reservation.
